# ICTV Virus Taxonomy Profile: *Globuloviridae*

**DOI:** 10.1099/jgv.0.001123

**Published:** 2018-08-09

**Authors:** David Prangishvili, Mart Krupovic, ICTV Report Consortium

**Affiliations:** Department of Microbiology, Institut Pasteur, 25 Rue du Dr Roux, 75015 Paris, France

**Keywords:** *Globuloviridae*, ICTV Report, Taxonomy

## Abstract

The family *Globuloviridae* comprises enveloped viruses with linear, double-stranded DNA genomes of about 21–28 kbp. The virions are spherical with a diameter of 70–100 nm. No information is available about genome replication. Globuloviruses infect hyperthermophilic archaea belonging to the genera *Pyrobaculum* and *Thermoproteus*, which thrive in extreme geothermal environments. Infection does not cause lysis of host cells and is noncytocidal. The viral genome does not integrate into the host chromosome. This is a summary of the International Committee on Taxonomy of Viruses (ICTV) Report on the taxonomy of the *Globuloviridae*, which is available at www.ictv.global/report/globuloviridae.

## Virion

**Fig. 1.**
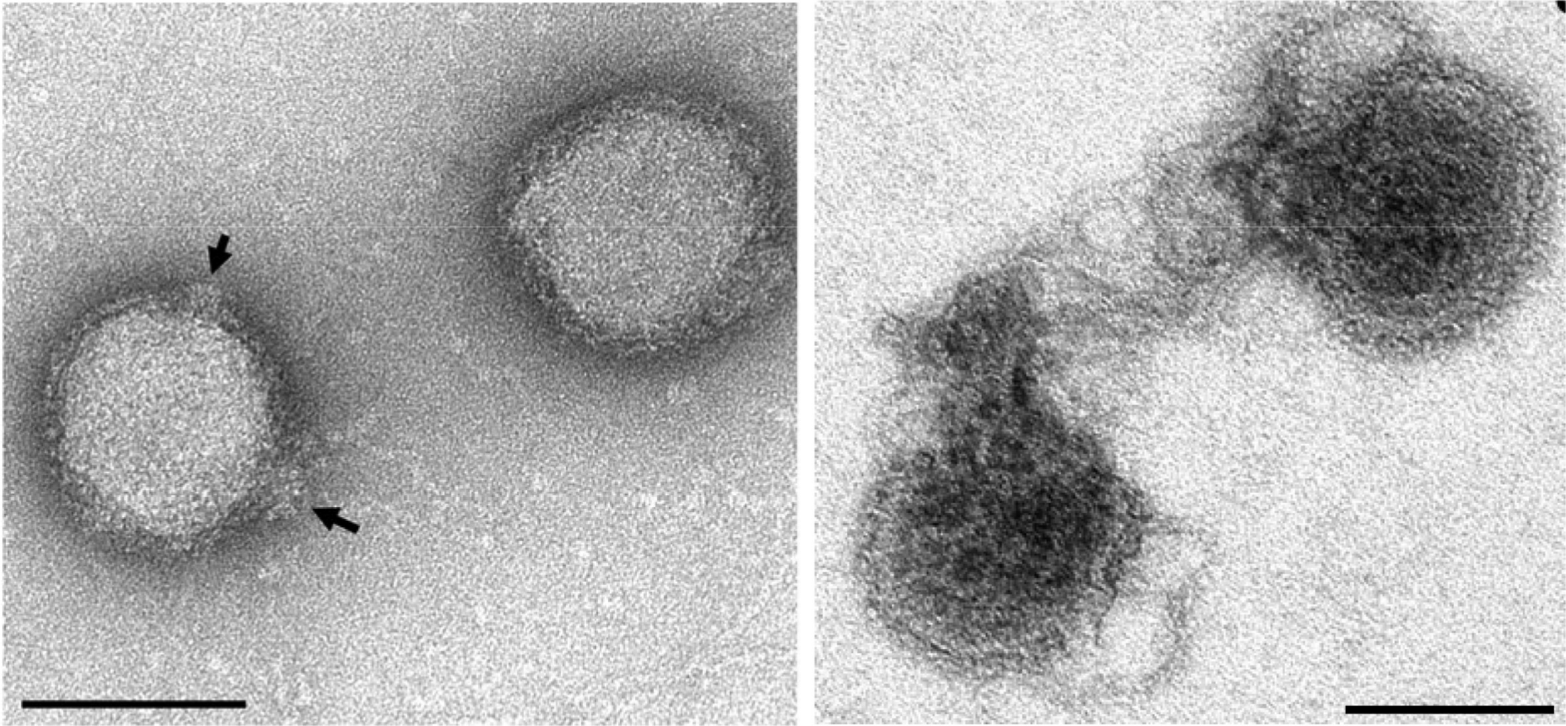
Negative-contrast electron micrographs of virions of Pyrobaculum spherical virus. (Left) Intact virions; arrows indicate spherical protrusions. (Right) Partially disrupted virions extruding helical nucleoprotein core. The bars represent 100 nm (modified from [1]).

Virions are spherical, 70–100 nm in diameter, with spherical protrusions that are about 15 nm in diameter (Table 1, Fig. 1). Virions carry a lipid-containing envelope that encases a superhelical core, consisting of linear dsDNA and three major structural proteins [1, 2]. The morphotype is unusual for dsDNA viruses [3, 4].

**Table 1.** Characteristics of the family *Globuloviridae*

Typical member:	Pyrobaculum spherical virus (AJ635161), species *Pyrobaculum spherical virus*, genus *Globulovirus*
Virion	Spherical, with a diameter of 70–100 nm; envelope encases helical nucleoprotein core
Genome	Linear, dsDNA genomes of about 21–28 kbp
Replication	Non-lytic, chronic infection
Translation	No information
Host range	Hyperthermophilic archaea from the genera *Pyrobaculum* and *Thermoproteus*
Taxonomy	One genus, two species

## Genome

**Fig. 2.**
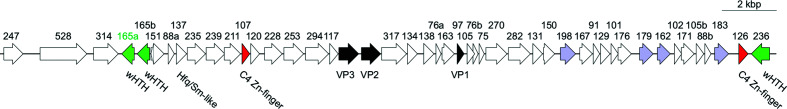
Genome organization of Pyrobaculum spherical virus showing location, sizes and transcriptional direction of putative genes. Genes encoding the three major structural proteins, VP1–3, are shown in black. Paralogous genes are indicated with the same colours. wHTH; gene encoding winged helix-turn-helix domain. Figure modified from [6].

The virion contains a single molecule of linear dsDNA, comprising 28 337 bp for Pyrobaculum spherical virus and about 21.6 kbp for Thermoproteus tenax spherical virus 1 [1, 2]. The ends of the linear genome carry inverted repeats (190 bp for Pyrobaculum spherical virus), which contain multiple copies of 5 bp direct repeats. In the case of Pyrobaculum spherical virus, the two strands of the dsDNA genome appear to be covalently linked at the termini [1]. Pyrobaculum spherical virus and Thermoproteus tenax spherical virus 1 genomes have 48 and 38 open reading frames (ORF), respectively, of which only 15 are shared between the two viruses. Almost all of the predicted genes are located on one DNA strand (Fig. 2) and do not show sequence similarity to genes in existing databases [5]. Several examples of gene duplication have been reported (Fig. 2). High-resolution structures for five Pyrobaculum spherical virus proteins have been solved by X-ray crystallography [6].

## Replication

Globuloviruses establish a chronic infection and are released from the host cells without causing lysis. The viruses do not encode identifiable genome replication proteins and are likely to recruit the host machinery for genome replication. Pyrobaculum spherical virus infects hyperthermophilic archaea from the genera *Pyrobaculum* and *Thermoproteus* [1], whereas Thermoproteus tenax spherical virus 1 has been shown to replicate in a single strain of *Thermoproteus* [2].

## Taxonomy

One genus, *Globulovirus*, with two species, *Pyrobaculum spherical viru*s and *Thermoproteus tenax spherical virus 1*. Viruses from these two species share less than half of their genes [1, 2]. Members of different species in the genus are distinguished by their host range and nucleotide sequence.

## Resources

Full ICTV Online (10th) Report: www.ictv.global/report/globuloviridae.
